# Bidirectional Mendelian randomization explores the causal relationship between dietary habits and rheumatoid arthritis

**DOI:** 10.1097/MD.0000000000039779

**Published:** 2024-09-20

**Authors:** Wantong Xu, Minghe Ouyang, Dan Peng, Zhongbiao Jiang

**Affiliations:** a Department of Orthopaedic Surgery, The Second Xiangya Hospital, Central South University, Changsha, China; b Xiangya School of Medicine, Central South University, Changsha, Hunan, China; c Department of Radiology, The Second Xiangya Hospital, Central South University, Changsha, China.

**Keywords:** dietary habits, Mendelian randomization, reverse Mendelian randomization, rheumatoid arthritis

## Abstract

Epidemiological and other studies have shown that the occurrence and progression of rheumatoid arthritis (RA) are closely related to diet. To further explore the causal association between dietary habits and RA, we performed a bidirectional Mendelian randomization (MR) analysis. The dataset related to dietary habits is from genome-wide association studies, including 143 dietary habits. The dataset of RA is from the FinnGen database. Inverse variance weighted (IVW), MR-Egger, simple mode, weighted median, and weighted mode were used for the 2-sample, 2-way MR analysis. At the same time, a variety of pleiotropic and heterogeneity tests were used to ensure the accuracy of the results. IVW results show that among current drinkers (drinks usually with meals yes + it varies vs no) was positively correlated with RA (β, 0.563 [95% confidence interval [CI], 0.286–0.840]; *P* = 6.7 × 10^−5^). Spread type (low fat spread vs any other) was negatively correlated with RA (β, −2.536 [95% CI, −3.725 to −1.346]; *P* = 2.9 × 10^−5^). In addition, the reverse MR results showed that RA was positively correlated with milk type (skimmed vs any other; β, 0.006 [95% CI, 0.000–0.011]; *P* = 4.5 × 10^−2^). RA was positively correlated with spread type (tub margarine vs never; β, 0.016 [95% CI, 0.002–0.029]; *P* = 2.5 × 10^−2^). The results of pleiotropy and heterogeneity tests showed that there was no pleiotropy (*P* > .05) in the obtained results. The analysis results of MR-Egger, simple mode, weighted median, and weighted mode are consistent with our IVW results. This study reveals a potential association between specific dietary habits and RA. Among current drinkers (drinks usually with meals yes + it varies vs no) was positively correlated with RA. Spread type (low fat spread vs any other) was negatively correlated with RA. RA was positively correlated with milk type (skimmed vs any other) and spread type (tub margarine vs never).

## 1. Introduction

Rheumatoid arthritis (RA) is a chronic systemic autoimmune disease. Its main pathological feature is the inflammation of joint synovium, accompanied by the progressive destruction of bone and cartilage. Patients often present with pain, swelling, and dysfunction of multiple joints, which can be accompanied by a variety of complications.^[1]^ In the past, it was generally believed that RA was mainly caused by genetic and environmental factors.^[2]^ However, some studies in recent years have shown that dietary habits are closely related to the occurrence and progression of RA.^[3]^ For example, a survey of 217 subjects showed that diet had an impact on the symptom performance of nearly 1/4 of RA subjects.^[4]^ In addition, the Mediterranean diet, as a dietary pattern considered relatively healthy, has also been reported to have a certain protective effect on RA.^[5]^ Therefore, understanding the influence of dietary habits on RA has positive significance for the clinical treatment and early prevention of RA. However, there are still few Mendelian randomization (MR) studies on the causal relationship between dietary habits and RA. Therefore, we performed this MR analysis to reveal the potential association between diet and RA.

MR is an analysis method that uses genetic variation as instrumental variables (IVs) to infer the causal relationship between exposure and outcome by establishing the association between IVs and exposure and outcome. MR analysis is based on 3 key assumptions: IVs are closely related to exposure, no association between IVs and confounders was observed, and the effect of IVs on outcomes occurred only through exposure. MR analysis has a large sample size and can reduce the interference of confounding factors on the results, which has obvious advantages over traditional epidemiological research. Therefore, this study used bidirectional MR to explore the causal association between dietary habits and RA.

## 2. Materials and methods

### 2.1. Data sources

We obtained the genetic variation data related to dietary habits from the genome-wide association study from the UK Biobank, which contains 143 dietary habits, including drinking, eggs, grains, sugar and salt intake, fruits, milk, cream, drinks, meat, and other common dietary factors. We sorted and selected 83 phenotypically clear dietary habits for our study. All RA data were obtained from the FinnGen database (https://r10.finngen.fi/), including 13,621 cases and 262,844 controls. All individuals were from European populations.

### 2.2. Instrumental variable

We first screened single-nucleotide polymorphisms (SNPs) significantly associated with dietary habits from the pooled data at the genome-wide significance threshold (*P* < 5 × 10^−8^). However, at this genome-wide significance threshold, there are too few candidate SNPs for each dietary habit phenotype, which is not conducive to further MR analysis. Therefore, this study used *P* < 5 × 10^−6^ to screen SNPs related to dietary habits to include more IVS to better prove the results.^[6]^ After SNPs were screened, linkage disequilibrium (R^2^ < 0.001; window size = 10,000 kb) was removed to ensure that each SNP was independent of each other. At the same time, the bias of weak IVs was calculated by calculating the F value, and the IVs with F < 10 were eliminated to ensure the strong association between IV and exposure.^[7]^ In addition, the MR-Pleiotropy Residual Sum and Outlier (MR-PRESSO) test was used to identify potential outliers and horizontal pleiotropy (*P* < .05). The abnormal SNPs were removed after being detected.

### 2.3. Statistical analysis

In this study, bidirectional MR analysis was conducted on 2 samples. The SNPs associated with dietary habits obtained after screening were used as IVs in the forward MR analysis, and RA was used as the outcome. The reverse MR analysis used SNPs associated with RA as IVs and dietary habits as outcomes.

We chose the inverse variance weighted (IVW) method as the preferred MR analysis method to determine the causal effect of exposure on outcomes because the IVW model is the most powerful method to detect causality in 2-sample MR analysis.^[8]^ At the same time, other MR methods (including MR-Egger, simple mode, weighted median, and weighted mode) are used as supplements to verify the reliability of the results.

In the sensitivity analysis, we used MR-Egger and MR-PRESSO to detect the pleiotropy of the results, where *P* < .05 indicates that the results have pleiotropy. IVW test and MR-Egger were used to detect the heterogeneity of the results, and *P* < .05 indicated that the results were heterogeneous. As mentioned above, we used the MR-PRESSO method to detect and eliminate possible outliers and pleiotropy.

## 3. Result

All IVs used for forward and reverse MR analysis after screening are shown in Table S1, Supplemental Digital Content, http://links.lww.com/MD/N631, and Table S2, Supplemental Digital Content, http://links.lww.com/MD/N631. Finally, the results with *P* < .05 and the MR-PRESSO test excluding pleiotropy were selected as positive results. In sensitivity analysis, MR-Egger and MR-PRESSO analysis showed that there was no pleiotropy in all positive results (*P* > .05), and there was basically no heterogeneity in the IVW test and MR-Egger analysis (*P* > .05).

### 3.1. Influence of dietary habits on RA

**Figure 1.**
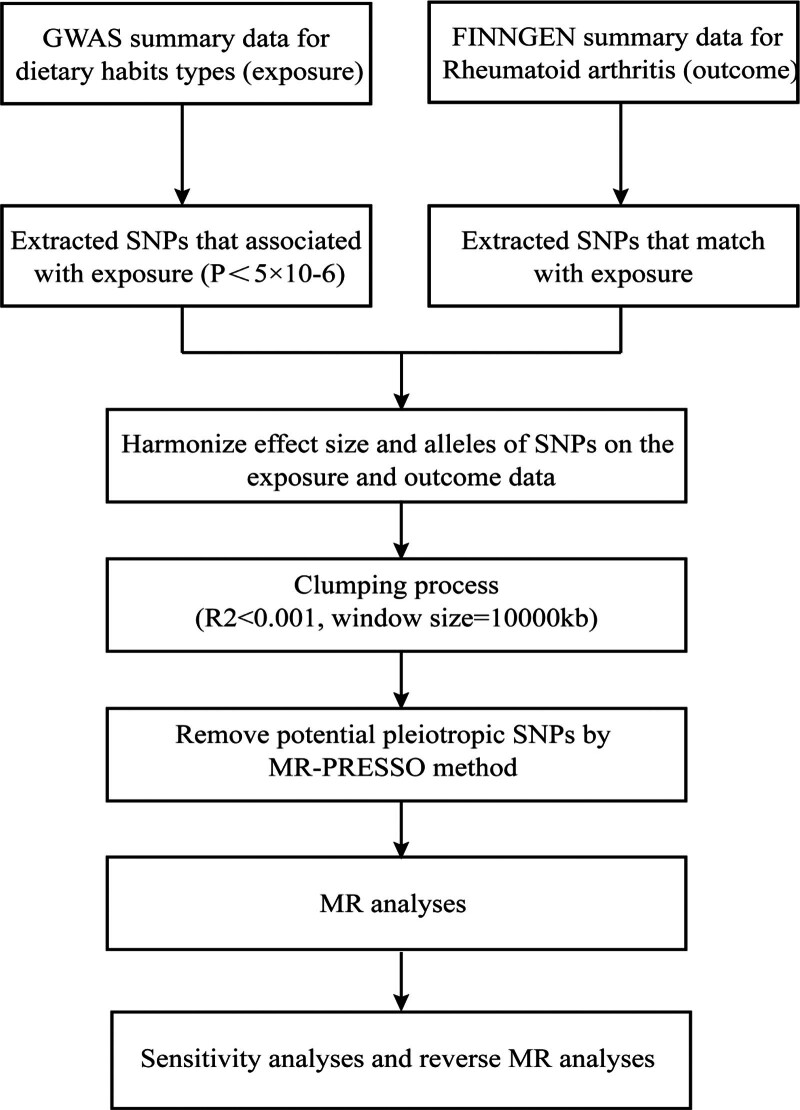
The working flow chart of this bidirectional 2-sample Mendelian randomization study to explore the causal relationship between rheumatoid arthritis and dietary habits. GWAS = genome-wide association study, MR = Mendelian randomization, PRESSO = Pleiotropy Residual Sum and Outlier, SNP = single-nucleotide polymorphism.

**Figure 2.**
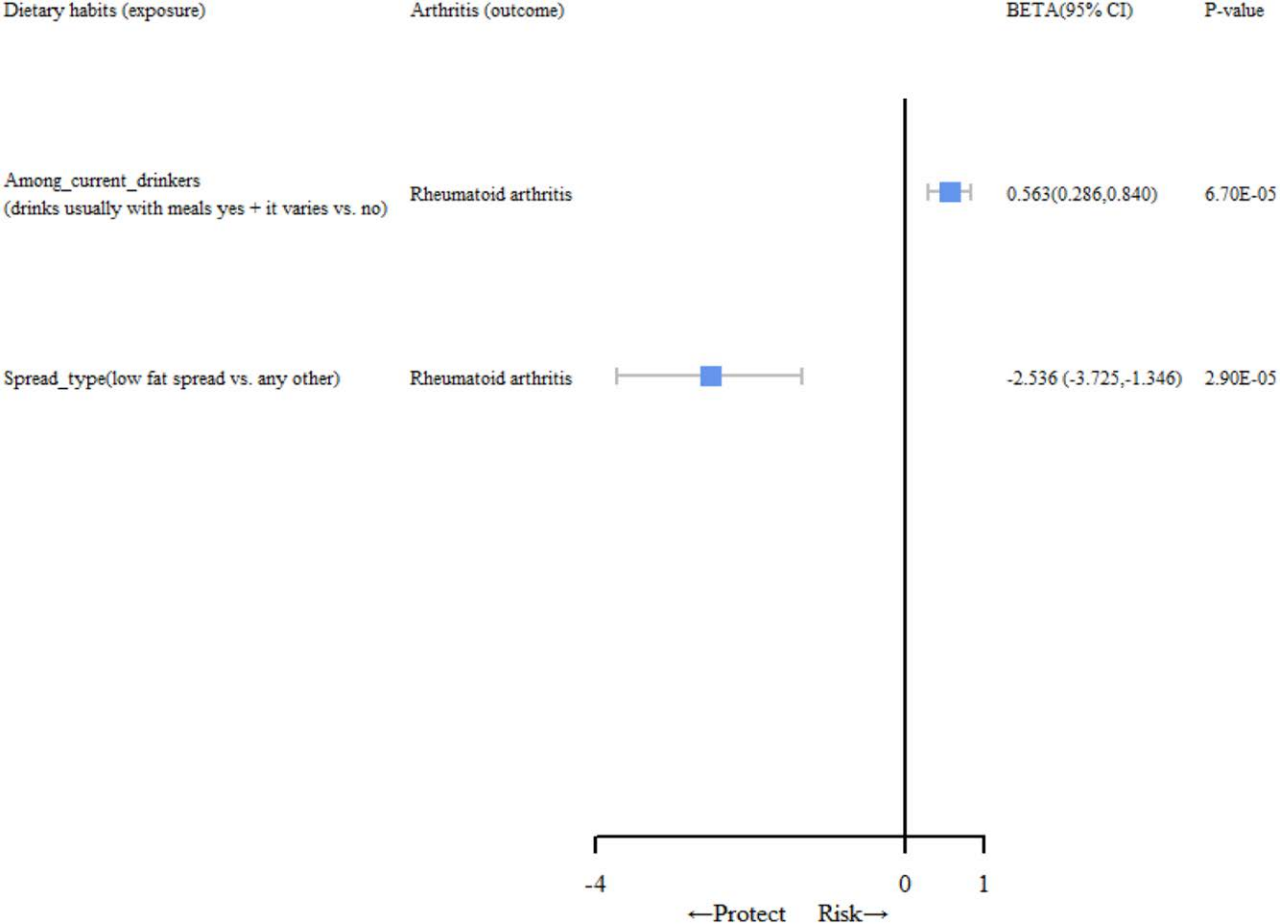
The forest plot shows the effect of specific dietary habits on rheumatoid arthritis in Mendelian randomization positive results. CI = confidence interval.

The workflow of the MR analyses conducted is succinctly depicted in Figure 1. The complete results of MR estimates for the causal association between dietary habits and RA are shown in Table S3, Supplemental Digital Content, http://links.lww.com/MD/N631. In the forward MR analysis, 2 positive results were obtained. As shown in Figure 2, IVW results show that among current drinkers (drinks usually with meals yes + it varies vs no) was positively correlated with RA (β, 0.563 [95% confidence interval [CI], 0.286–0.840]; *P* = 6.7 × 10^−5^). Spread type (low fat spread vs any other) was negatively correlated with RA (β, −2.536 [95% CI, −3.725 to −1.346]; *P* = 2.9 × 10^−5^). The results estimated by other supplementary MR methods are shown in Table 1. As depicted in Table S5.1, Supplemental Digital Content, http://links.lww.com/MD/N631, no reverse causal relationship was observed among the associations identified through the aforementioned MR analysis.

**Table 1:** Full positive results of forward MR estimate for the association between dietary habit types and arthritis.

Dietary habits (exposure)	Arthritis (outcome)	MR method	No. SNP	F	β (95% CI)	*P* value
Among_current_drinkers (drinks usually with meals yes + it varies vs no)	Rheumatoid arthritis	IVW (MRE)	89	22.03	0.563 (0.286–0.840)	6.70 × 10^−5^
		MR-Egger	89		0.335 (−0.643 to 1.313)	.5
		Weighted median	89		0.407 (0.044 to 0.771)	2.80 × 10^−2^
		Simple mode	89		0.349 (−0.610 to 1.309)	.48
		Weighted mode	89		0.371 (−0.463 to 1.206)	.39
Spread_type (low fat spread vs any other)	Rheumatoid arthritis	IVW (MRE)	15	24.44	−2.536 (−3.725 to −1.346)	2.90 × 10^−5^
		MR-Egger	15		−2.238 (−4.585 to 0.110)	.08
		Weighted median	15		−2.896 (−4.486 to −1.305)	3.60 × 10^−4^
		Simple mode	15		−3.451 (−6.189 to −0.713)	2.70 × 10^−2^
		Weighted mode	15		−3.558 (−6.710 to −0.405)	4.40 × 10^−2^

CI = confidence interval, IVW = inverse variance weighted, MR = Mendelian randomization, MRE = multiplicative random-effects model, SNP = single-nucleotide polymorphism.

A total of 6 causal effects were identified from dietary habits to RA. Of these, the F-statistic of the IVs ranged from 22.03 to 24.44, suggesting that there was no weak IV bias, as shown in Table 1. The results of the IVW test and MR-Egger showed no significant heterogeneity in these IVs. Besides, the MR-Egger intercept and MR-PRESSO global test were utilized to test for horizontal pleiotropy, and all *P*-values were >0.05, indicating no significant directional horizontal pleiotropy, as shown in Table 2 and Table S6, Supplemental Digital Content, http://links.lww.com/MD/N631. The complete results of the sensitivity analysis are shown in Table S8.1, Supplemental Digital Content, http://links.lww.com/MD/N631.

**Table 2:** Sensitivity analyses of forward MR for association between dietary habit types and arthritis.

Dietary habits (exposure)	Arthritis (outcome)	No. SNP	Pleiotropy		Heterogeneity	
			MR-PRESSO global *P* value	MR-Egger *P* value	IVW test *P* value	MR-Egger *P* value
Among_current_drinkers (drinks usually with meals yes + it varies vs no)	Rheumatoid arthritis	89	.058	.634724174	.07323807	.06593410
Spread_type (low fat spread vs any other)	Rheumatoid arthritis	15	.1576	.774868995	.27750251	.22304553

MR = Mendelian randomization, PRESSO = Pleiotropy Residual Sum and Outlier, SNP = single-nucleotide polymorphism.

### 3.2. Influence of RA on dietary habits

**Figure 3.**
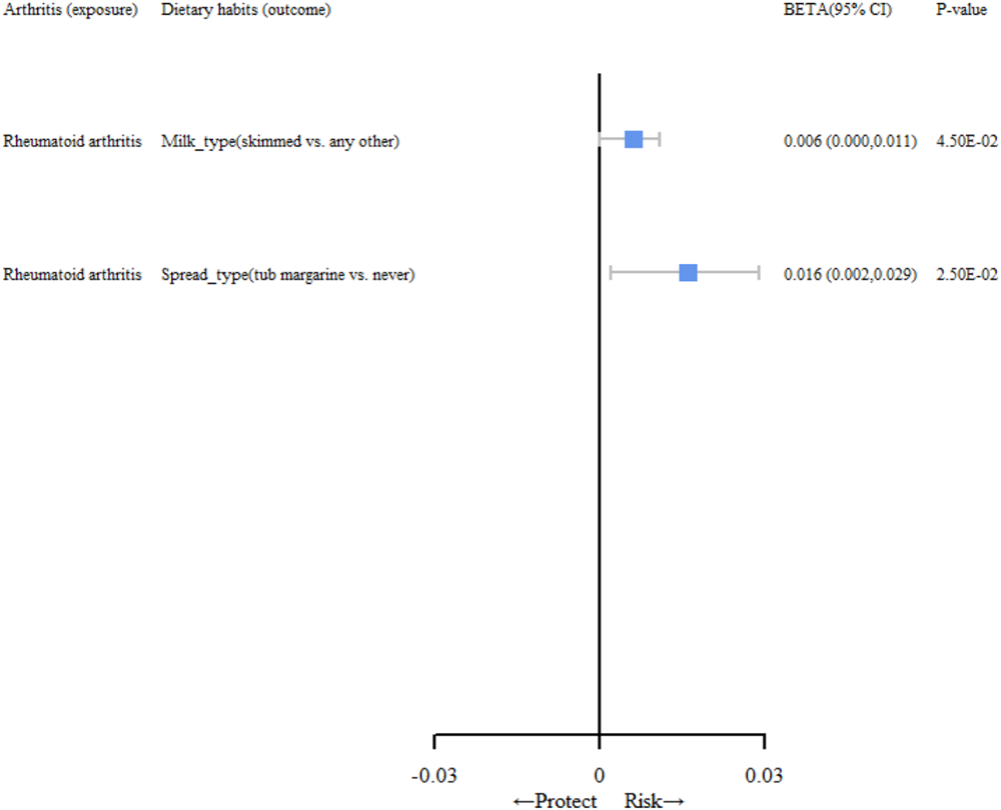
The forest plot shows the effect of specific rheumatoid arthritis on dietary habits in Mendelian randomization positive results. CI = confidence interval.

To deeply explore the causal effects of RA on dietary habits, a bidirectional MR analysis was conducted. As shown in Figure 3, the reverse MR results showed that RA was positively correlated with milk type (skimmed vs any other; β, 0.006 [95% CI, 0.000–0.011]; *P* = 4.5 × 10^−2^). RA was positively correlated with spread type (tub margarine vs never; β, 0.016 [95% CI, 0.002–0.029]; *P* = 2.5 × 10^−2^). The results estimated by other supplementary MR methods are shown in Table 3. The complete results of MR estimates for the causal association between RA and dietary habits are shown in Table S4, Supplemental Digital Content, http://links.lww.com/MD/N631. As shown in Table S5.2, Supplemental Digital Content, http://links.lww.com/MD/N631, no positive causality was found in the associations identified by the reverse MR analysis. Furthermore, the conducted sensitivity analysis (Table 4 and Table S8.2, Supplemental Digital Content, http://links.lww.com/MD/N631) suggested that any potential horizontal pleiotropy was improbable to distort the established causal relationship between the aforementioned factors. The complete results of the sensitivity analysis are shown in Table S7, Supplemental Digital Content, http://links.lww.com/MD/N631. Funnel and scatter plots and forest plots illustrating the relationship between dietary habits and RA are shown in Figures 1 to 3, Supplemental Digital Content, http://links.lww.com/MD/N630, respectively. The analysis results of MR-Egger, simple mode, weighted median, and weighted mode are consistent with our IVW results.

**Table 3:** Full positive results of inverse MR estimate for the association between arthritis and dietary habit types.

Arthritis (exposure)	Dietary habits (outcome)	MR method	No. SNP	F	β (95% CI)	*P* value
Rheumatoid arthritis	Milk_type (skimmed vs any other)	IVW (MRE)	61	22.02	0.006 (0.000 to 0.011)	4.50 × 10^−2^
		MR-Egger	61		0.009 (0.001 to 0.017)	4.10 × 10^−2^
		Weighted median	61		0.010 (0.002 to 0.018)	1.20 × 10^−2^
		Simple mode	61		0.003 (−0.016 to 0.023)	0.74
		Weighted mode	61		0.009 (0.001 to 0.018)	3.90 × 10^−2^
Rheumatoid arthritis	Spread_type (tub margarine vs never)	IVW (MRE)	61	22.02	0.016 (0.002 to 0.029)	2.50 × 10^−2^
		MR-Egger	61		0.006 (−0.015 to 0.027)	0.58
		Weighted median	61		0.007 (−0.014 to 0.027)	0.53
		Simple mode	61		0.011 (−0.027 to 0.048)	0.59
		Weighted mode	61		0.005 (−0.014 to 0.024)	0.59

CI = confidence interval, IVW = inverse variance weighted, MR = Mendelian randomization, MRE = multiplicative random-effects model, SNP = single-nucleotide polymorphism.

**Table 4:** Sensitivity analyses of inverse MR for association between arthritis and dietary habit types.

Arthritis (exposure)	Dietary habits (outcome)	No. SNP	Pleiotropy		Heterogeneity	
			MR-PRESSO Global *P* value	MR-Egger *P* value	IVW test *P* value	MR-Egger *P* value
Rheumatoid arthritis	Milk_type (skimmed vs any other)	61	.0546	.299003729	.0412660	.04336713
Rheumatoid arthritis	Spread_type (tub margarine vs never)	61	.1768	.256976568	.17908882	.18869211

IVW = inverse variance weighted, MR = Mendelian randomization, PRESSO = Pleiotropy Residual Sum and Outlier, SNP = single-nucleotide polymorphism.

## 4. Discussion

The potential effect of diet on RA has been a hot topic in epidemiological and immunological research. To the best of our knowledge, this is the first study using the MR method to explore the causal relationship between RA and specific dietary habits on a large scale. The detailed pathway by which diet affects RA is not clear, but a large number of studies have shown that gut microbiota may play an important intermediate bridge role in it.^[9]^ The gut microbiota includes all commensal bacteria and potentially pathogenic bacteria in the human gastrointestinal system, the vast majority of which belong to bacteroidetes and firmicutes.^[10]^ It is worth noting that both prevotella and bacteroidetes are quite sensitive to diet. Studies have found that the increase in prevotella is associated with a high-fiber diet, while the increase in bacteroidetes is associated with a diet rich in fat and animal protein.^[11]^ In addition, human studies have shown that there are significant differences in the intestinal microbiota of patients with RA compared with healthy controls, and the intestinal microbial diversity is reduced, which is characterized by a lower bacterial abundance of bifidobacterium and bacteroidetes families, while after patients use antirheumatic drugs, the altered microbiota can be partially restored.^[12]^ Combined with our results, it can be speculated that different dietary habits may ultimately promote the occurrence and development of distant inflammation, such as arthritis, by affecting the abundance and metabolic changes of gut microbiota.

**Figure 4.**
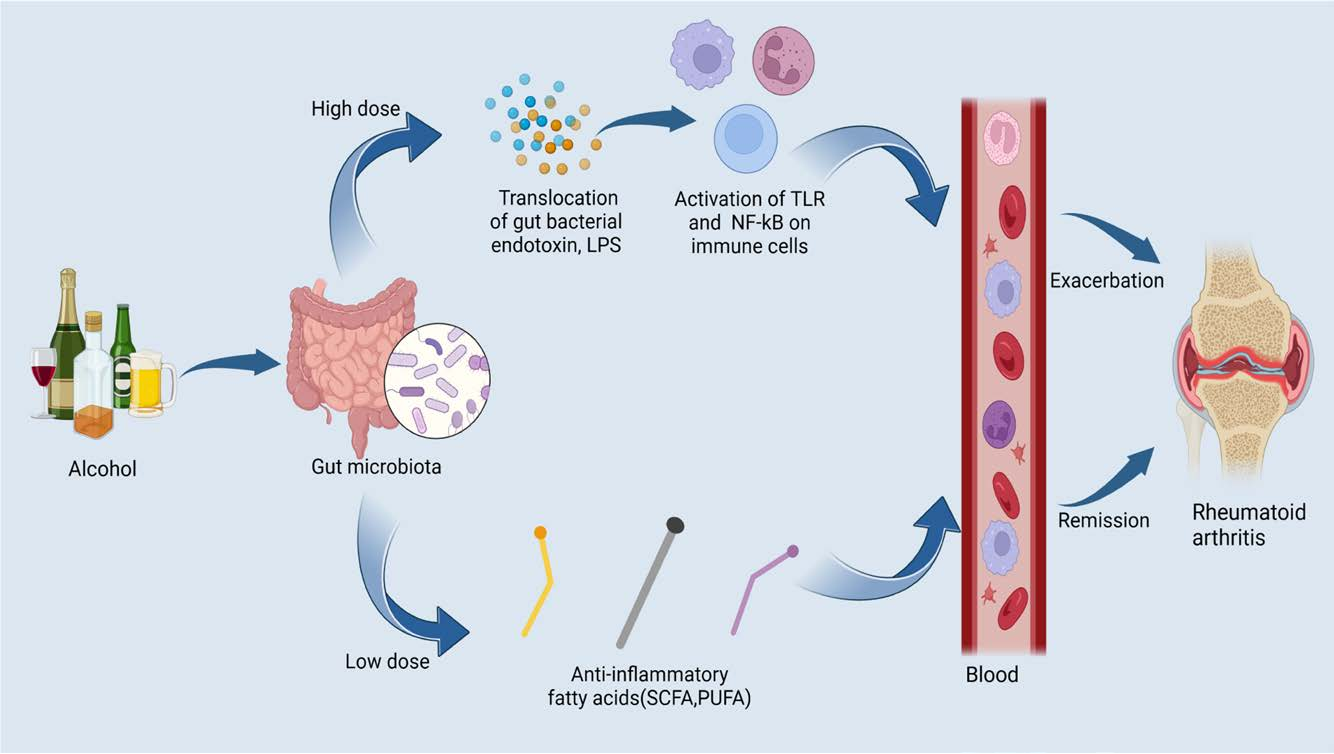
The potential mechanism of dual effects of alcohol on rheumatoid arthritis (RA). High doses of alcohol have been found to cause dysbiosis of the gut microbiome. Microbiome dysbiosis can lead to impaired intestinal permeability and promote inflammation via systemic translocation of gut bacterial endotoxin, lipopolysaccharide (LPS), activation of toll-like receptor (TLR), and nuclear factor-κB (NF-kB) on immune cells, while low-to-moderate doses of alcohol may help the gut produce derived anti-inflammatory fatty acids, such as short-chain fatty acids (SCFAs) and polyunsaturated fatty acids (PUFAs), which play a certain protective role against RA. Figure was created with BioRender (https://biorender.com).

Drinking alcohol is a widespread dietary practice. In fact, the effects of alcohol on autoimmune diseases such as RA are quite complex. Long-term high-dose drinking may promote the progression of inflammation, leading to an increased risk of RA and further deterioration.^[13]^ This is consistent with our analysis of MR. However, the study found that mild to moderate alcohol intake had a certain protective effect on RA, which showed a J- or U-shaped dose dependence.^[14]^ In experimental studies, moderate alcohol consumption has been shown to reduce the incidence of collagen-induced arthritis.^[15]^ Several systematic reviews and meta-analyses also found that compared with nondrinkers, moderate drinkers have lower RA risk, lower disease activity, and better quality of life.^[16,17]^ In addition, given that alcohol is mainly metabolized in the gastrointestinal tract, the effect of alcohol on the gut microbiota is equally noteworthy. High doses of alcohol have been found to cause dysbiosis of the gut microbiome. As shown in Figure 4, microbiome dysbiosis can lead to impaired intestinal permeability and promote inflammation via systemic translocation of gut bacterial endotoxin, lipopolysaccharide, activation of toll-like receptors, and nuclear factor-κB on immune cells,^[18]^ while low-to-moderate doses of alcohol may help the gut produce derived anti-inflammatory fatty acids, such as short-chain fatty acids and polyunsaturated fatty acids, which play a certain protective role against RA.^[19]^ Therefore, our MR results may only be a preliminary conclusion, and further MR research for quantitative analysis of alcohol dose is very necessary.

Fat intake is another important dietary factor. MR analysis showed that spread type (low fat spread vs any other) was negatively correlated with RA. Low-fat intake may be more beneficial for RA. This result is basically consistent with previous studies. In another MR study exploring the causal relationship between body fat composition and RA, the results showed a significant causal relationship between fat mass and RA risk.^[20]^ Clinically, it was found that patients with RA with a high-fat diet had more severe symptoms than patients with ordinary RA, and anti-TNF-α treatment of RA is often ineffective in patients with obesity.^[21]^ It is worth noting that patients with RA often have abnormal lipid metabolism, and these abnormal lipid metabolites can also affect the progression of RA.^[22]^ For example, lipid abnormalities in T cells in patients with RA are thought to contribute to tissue invasion and migration, promote synovial inflammation, and erode cartilage and bone.^[23]^ Therefore, the relationship between high-fat intake and abnormal lipid metabolic pathways may need further exploration. Given that high-fat intake is a risk factor for RA, and skimmed milk and margarine tend to have lower fat content compared with their conventional counterparts, patients with RA may make some changes to their dietary habits. This may be a potential explanation for the reverse MR results that we observed.

Our study offers the following advantages. Compared to traditional epidemiological methods, MR analysis boasts a larger sample size, effectively minimizing the impact of reverse causality and potential confounding factors on results. The study also employed various sensitivity analysis methods to ensure the robustness of our findings. However, this research also has some limitations. The data used in this study are all from European populations, and the results obtained may not be applicable to other populations. In addition, this study did not consider the potential impact of complex dietary habits on RA, only examining the causal relationship between individual dietary habits and RA. Finally, this study did not include age, gender, or other factors, that may be significant. For instance, a study in the Chinese population found that increased alcohol consumption was associated with an elevated risk of RA in women but not in men.^[24]^ This study also proposed some follow-up valuable research directions, such as the specific impact mechanism of alcohol dose on RA, and whether the abnormal lipid metabolism of patients with RA is related to high-fat intake.

## 5. Conclusion

Based on the MR analysis, this study revealed the causal association between dietary habits and RA. Among current drinkers (drinks usually with meals yes + it varies vs no) was positively correlated with RA. Spread type (low fat spread vs any other) was negatively correlated with RA. The reverse MR results showed that RA was positively correlated with milk type (skimmed vs any other) and spread type (tub margarine vs never). Through this study, we further confirmed that fat intake is a risk factor for RA and provided new credible evidence for the complex effect of alcohol on RA. However, how to prevent or treat RA from the perspective of dietary habits still needs further research and exploration.

## Acknowledgments

The consortium’s release of dietary habits phenotypes of the T2DKP Summary Statistic was appreciated by the authors. The authors also wish to thank the authors of the genome-wide association study summary statistics for rheumatoid arthritis from FinnGen.

## Author contributions

**Data curation:** Wantong Xu.

**Formal analysis:** Wantong Xu.

**Methodology:** Wantong Xu, Minghe Ouyang.

**Visualization:** Minghe Ouyang, Dan Peng, Zhongbiao Jiang.

**Writing – original draft:** Minghe Ouyang.

**Conceptualization:** Dan Peng.

**Project administration:** Dan Peng, Zhongbiao Jiang.

**Software:** Dan Peng.

**Funding acquisition:** Zhongbiao Jiang.

**Resources:** Zhongbiao Jiang.

**Writing – review & editing:** Zhongbiao Jiang.

## Supplementary Material


