# Supplementary material for: Bidirectional Mendelian randomization explores the causal relationship between dietary habits and rheumatoid arthritis
Source: Medicine (Baltimore). 2024 Sep 20;103(38):e39779. doi: 10.1097/MD.0000000000039779 (PMC11419428; doi:10.1097/MD.0000000000039779)

S-figure 1. Causal relationships between dietary habits tpyes and rheumatoid arthritis in funnel plots.

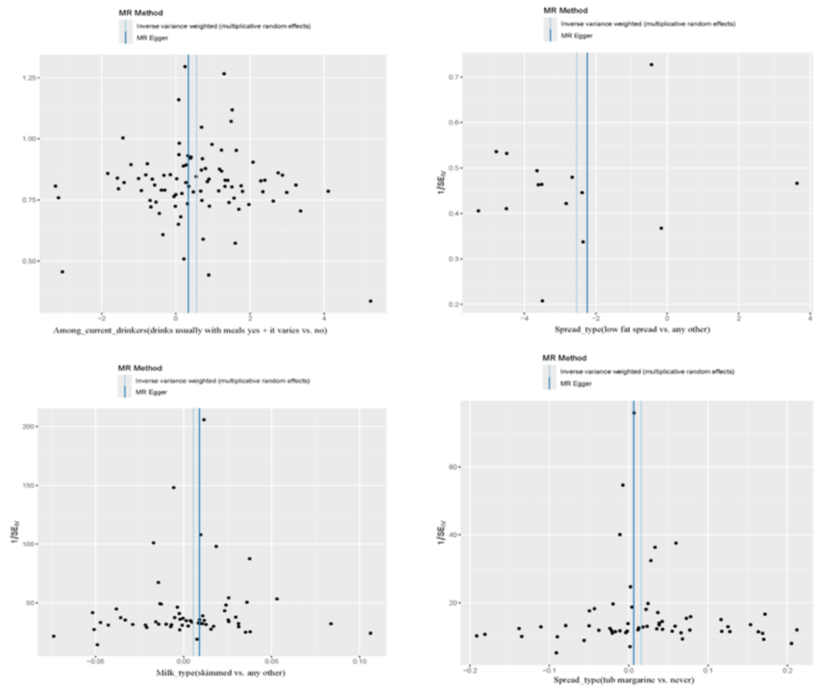

S-figure 2. Causal relationships between dietary habits tpyes and rheumatoid arthritis in scatter plots.

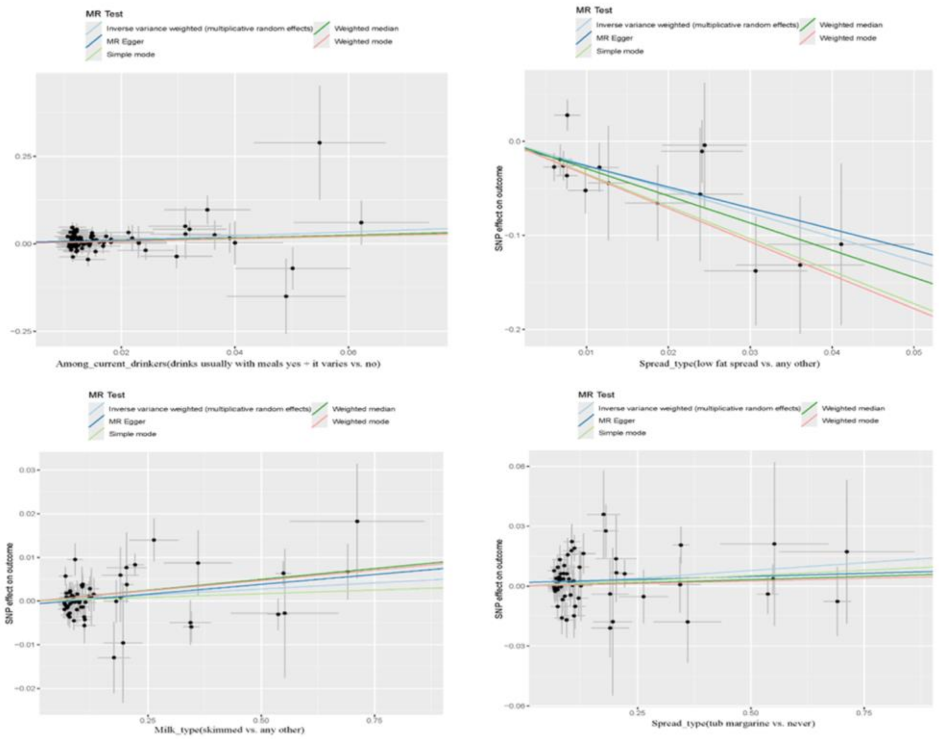

S-figure 3. Forest plots of SNPs associated with dietary habits tpyes and rheumatoid arthritis.

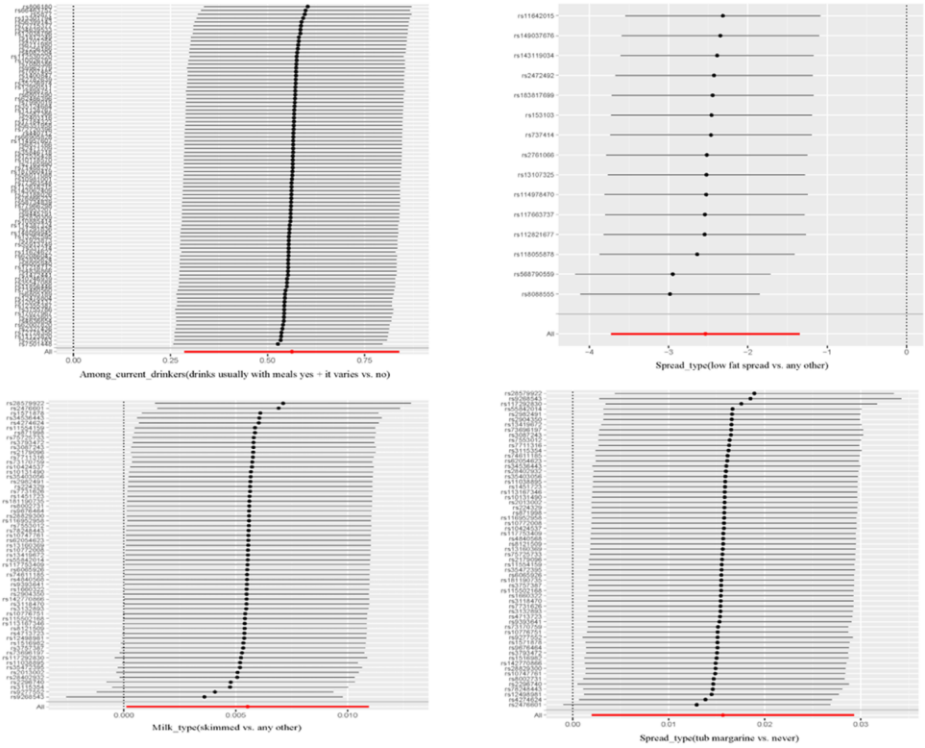

Supplement: Supplementary file 2 [file medi-103-e39779-s002.pdf]
